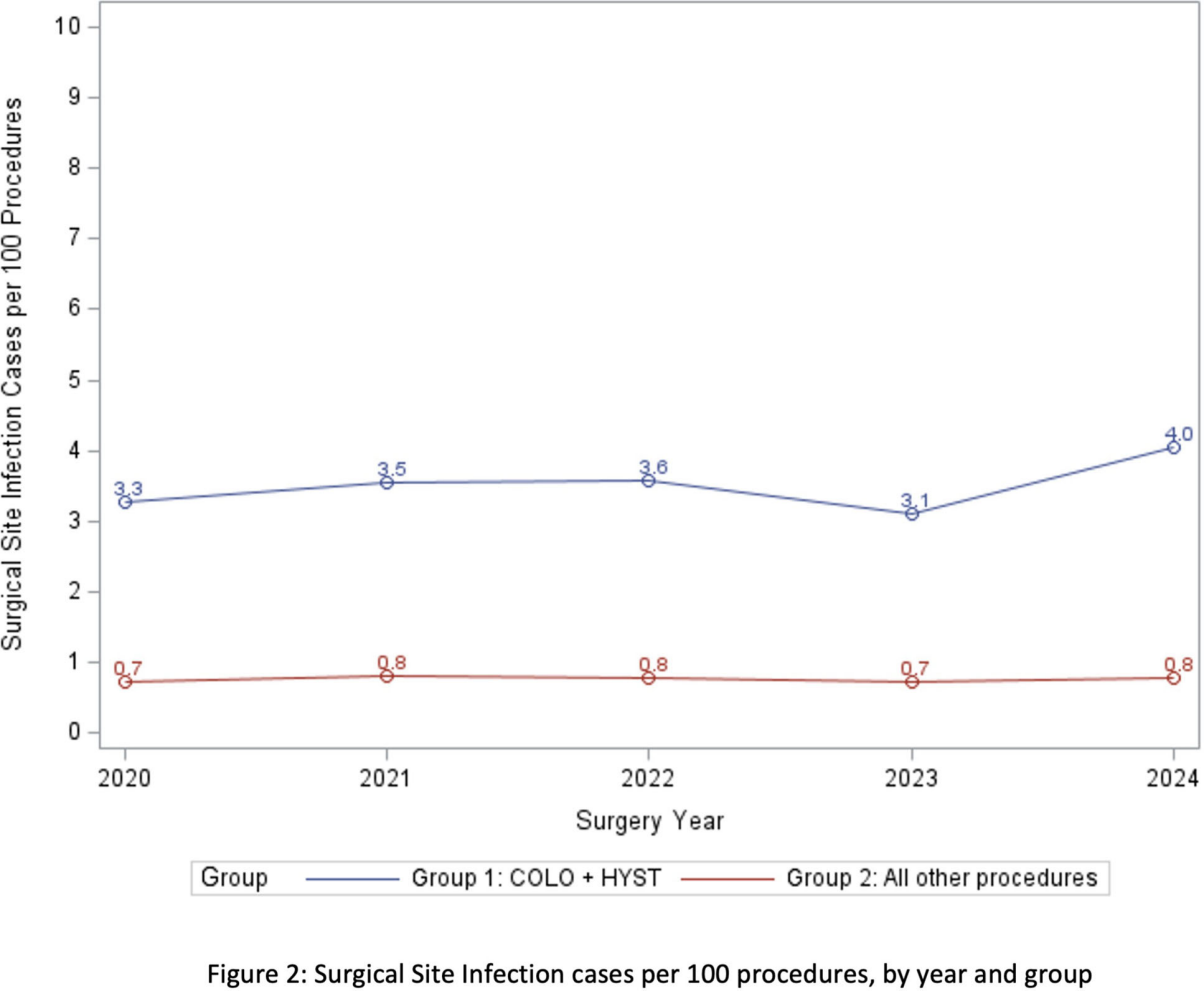# 57 Beyond Compliance: Achieving Sustained Excellence in Colorectal SSI Prevention Through Six Years of Bundled Prevention Strategies

**DOI:** 10.1017/ash.2026.10713

**Published:** 2026-06-23

**Authors:** Arielle Cohen, Brittain Wood, Polly Padgette, Valerie Payne, Linda Crane, Linda Roach, David Weber, Deverick Anderson, Jessica Seidelman

**Affiliations:** 1 Duke University; 2 Duke; 3 Duke Infection Control Outreach Network; 4 Duke University Medical Center; 5 Duke Ic Outreach Network; 6 University of North Carolina at Chapel Hill; 7 Duke Center for Antimicrobial Stewardship and Infection Prevention

## Abstract

**Background:** National Healthcare Safety Network (NHSN) data demonstrate substantial declines over time in most hospital-acquired infections (HAIs). In contrast, surgical site infection (SSI) rates have remained largely unchanged, raising concerns regarding the effectiveness of current prevention strategies. Public reporting and pay-for-performance programs primarily focus on Centers for Medicare & Medicaid Services (CMS)–mandated procedures, notably abdominal hysterectomy (HYST) and colon surgery (COLO). It remains unclear whether stagnant SSI rates reflect trends limited to these procedures or represent a broader phenomenon across surgical populations. We sought to determine whether temporal stagnation in SSI rates is confined to CMS-mandated procedures. Methods We conducted a retrospective cohort study using the Duke Infection Control Outreach Network (DICON) database, which includes standardized NHSN SSI surveillance data from participating hospitals. We included hospitals that were continuously enrolled in DICON for the entire study period (January 1, 2020–December 31, 2024). Procedures were excluded if a hospital performed fewer than 100 of a given procedure type in any calendar year. NHSN procedure categories were excluded entirely if <1,000 procedures were performed during the study period. Procedures were grouped as Group 1 (CMS-mandated HYST and COLO) and Group 2 (all other included procedures). Temporal trends in SSI rates were assessed using regression-based models with an interaction term between calendar year and procedure group, adjusting for age, sex, and wound class. Results A total of 64 hospitals met the inclusion criteria. The final dataset included 561,811 total procedures, of which 71,573 (12.7%) were in Group 1 (HYST/COLO) and 490,238 (87.3%) were in Group 2. SSI rates were 3.49 per 100 procedures in Group 1 and 0.77 per 100 procedures in Group 2. (Figure 1) Over the study period, annual SSI rates did not change significantly in Group 1 (annual rate ratio [aRR] 1.03; 95% CI 0.99–1.06, p=0.06) or Group 2 ([aRR] 1.01; 95% CI .98-1.03). (Figure 2) Moreover, there was no significant difference in annual SSI rates between COLO/HYST and other procedures (difference in annual trend aRR 0.98; 95% CI 0.94–1.02; p=0.25). Discussion In this multicenter analysis, stagnation in SSI rates was observed among both CMS-mandated procedures and non-mandated surgical procedures, suggesting that stable SSI rates reflect broader challenges in SSI prevention rather than artifacts related to surveillance or reporting. These findings highlight the need for novel, risk-adjusted prevention strategies.

**Figure:**
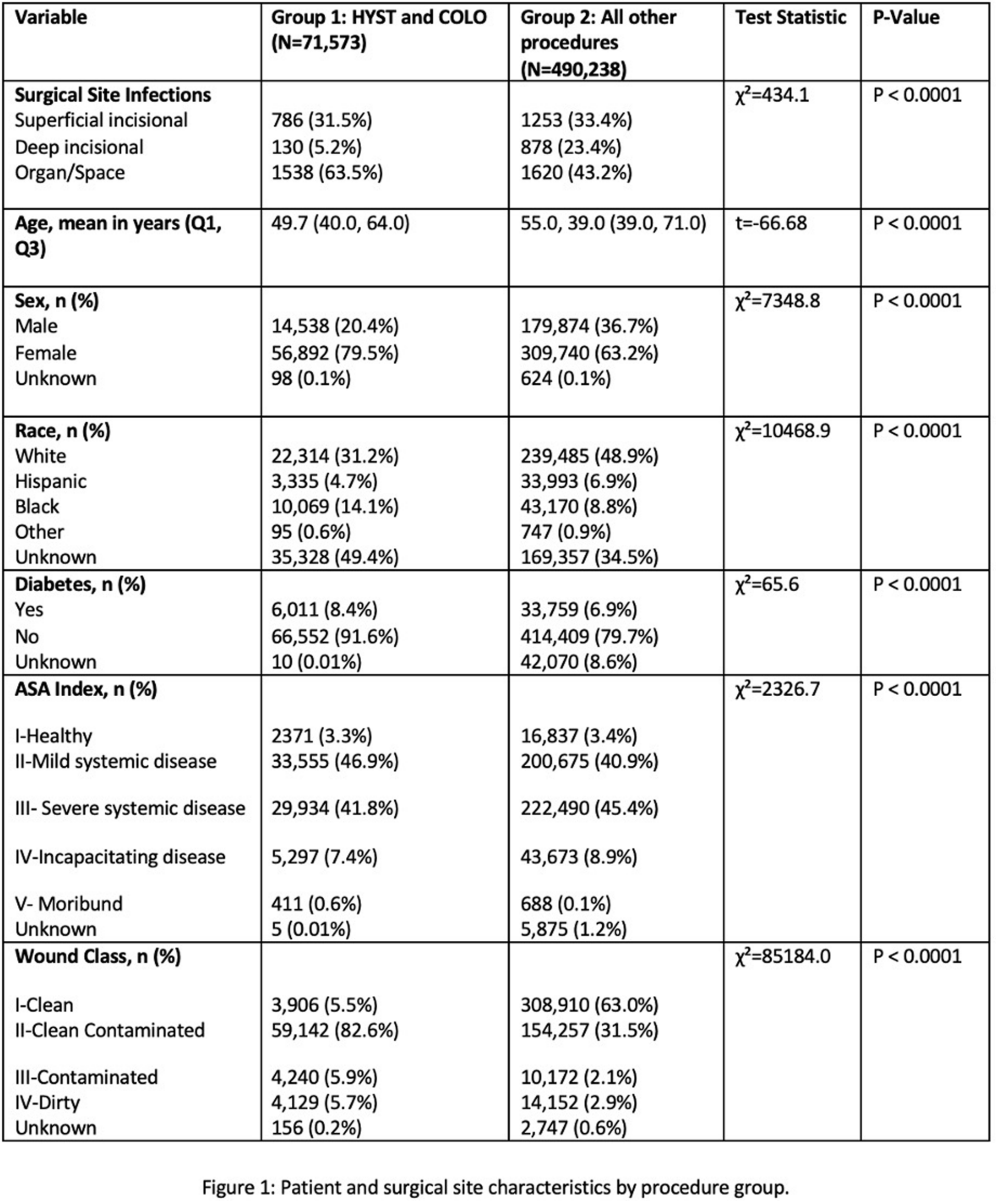

**Figure:**